# Landscape genomics and biased *FST *approaches reveal single nucleotide polymorphisms under selection in goat breeds of North-East Mediterranean

**DOI:** 10.1186/1471-2156-10-7

**Published:** 2009-02-19

**Authors:** Lorraine Pariset, Stephane Joost, Paolo Ajmone Marsan, Alessio Valentini, Econogene Consortium (EC)

**Affiliations:** 1 Dipartimento di Produzioni Animali, Università della Tuscia, Viterbo, Italy; 2 Istituto di Zootecnica, Università Cattolica del Sacro Cuore, Piacenza, Italy; 3 MFSA Research Center, Saint-Sulpice, Switzerland; 4 GIS Research Laboratory, Ecole Polytechnique Fédérale de Lausanne, Switzerland

## Abstract

**Background:**

In this study we compare outlier loci detected using a *FST *based method with those identified by a recently described method based on spatial analysis (SAM). We tested a panel of single nucleotide polymorphisms (SNPs) previously genotyped in individuals of goat breeds of southern areas of the Mediterranean basin (Italy, Greece and Albania). We evaluate how the SAM method performs with SNPs, which are increasingly employed due to their high number, low cost and easy of scoring.

**Results:**

The combined use of the two outlier detection approaches, never tested before using SNP polymorphisms, resulted in the identification of the same three loci involved in milk and meat quality data by using the two methods, while the *FST *based method identified 3 more loci as under selection sweep in the breeds examined.

**Conclusion:**

Data appear congruent by using the two methods for *FST *values exceeding the 99% confidence limits. The methods of *FST *and SAM can independently detect signatures of selection and therefore can reduce the probability of finding false positives if employed together. The outlier loci identified in this study could indicate adaptive variation in the analysed species, characterized by a large range of climatic conditions in the rearing areas and by a history of intense trade, that implies plasticity in adapting to new environments.

## Background

Population genomics relies on the principle that loci across the genome are influenced by genome-wide evolutionary force, whereas selection is locus-specific and imprints a particular pattern of variability only on linked loci [1,2]. Increasing attention has been put in understanding what proportion of a genome or which genes are being shaped by selection. Directional (Darwinian) selection can leave a set of signatures in the genes under its influence, such as the rapid divergence of functional sites among species and the depression of polymorphism within species. On the basis of these signatures, it is possible to identify genes or chromosomal regions which are likely targets of positive selection [3,4].

This is achieved by comparing all the loci across the genome that respond similarly to demography and neutral history of populations with outlier loci that show patterns of variation that deviate from the rest of the genome. Identification of outliers is important for two main reasons: i. such loci are potentially under selection and could be a sign of adaptive variation; ii. they could also bias estimates of population genetic parameters such as gene flow, population size and structure, and therefore should be excluded from these analyses. However, selected loci could be used in studies to better understand adaptation or to plan conservation-management strategies [5].

The identification of genes that have undergone positive selection is an important step in understanding how populations have adapted to environmental changes. Such studies are increasingly widespread [6-10] and their application to livestock species can also reveal insights on their selection history.

Aberrant behaviour of a locus can range from having exceptionally high or low *FST *between populations, to having an excess or deficit of low frequency alleles in a population [5].

Several statistical methods, in which loci candidate for selection are identified in the extreme tails of empirical distributions, have become a widely used strategy in genome-wide scans for selection [11-17]. However, several studies pointed out important methodological conditions to ensure the correct application of these methods [18] or stressed their lack of power [19-21].

*FST *statistic can be used to assess if the variation of SNP allele frequencies among populations leads to signatures of selection [2,22]. If *FST *is determined only by genetic drift, all loci across the genome are affected in a similar way. In the presence of locus-specific selection pressure, deviation in *FST *values is observed in selected loci and in linked genetic markers. See Table 3 for SNPs showing simulated *FST*.

In this study we want to compare outlier loci assessed using a *FST *based method [22] with those identified by a recently described method [8] based on spatial analysis (SAM). In the original paper Joost *et al*. tested the efficacy of SAM against *FST *based methods using AFLP and microsatellite data from two different species, showing a strong correspondence between the two approaches [8]. SAM is an implementation of logistic regression resembling the method applied by Jump *et al*. to plant populations with AFLP markers [23]; but it is used in an explorative way rather than for the confirmation of working hypotheses [8,24] and therefore the processing of all possible univariate association models between all environmental parameters and the presence or absence of all single alleles is carried out.

Here we evaluate how *FST *and SAM perform with a different kind of marker, single nucleotide polymorphisms (SNPs), which are increasingly employed due to their high number, low cost and easy of scoring. Besides, SNP can be found inside gene candidates for artificial or natural selection and therefore they might be more informative for this purpose rather than neutral or random markers, like microsatellites and AFLPs.

We analysed goat breeds of southern areas of the Mediterranean basin (Italy, Greece and Albania) sampled in the context of the European ECONOGENE Project  using a set of SNPs previously described [25]. See Table 2 for Breeds analysed, country of origin and their sample sizes (N).

## Results

**Figure 1:**
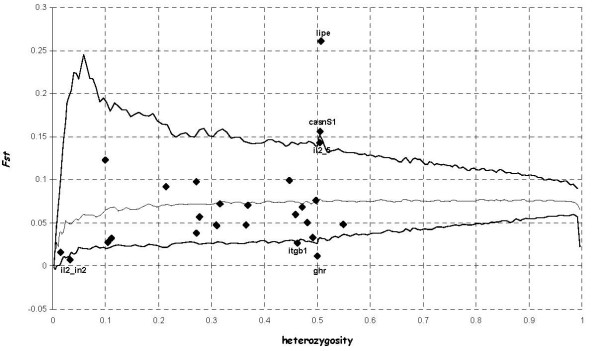
**Plot of *F*_*ST *_against heterozygosity for the 27 SNPs analysed**. Distribution of *F*_ST _values as a function of the within-population heterozygosity (*H*_S_) based on the 27 goat SNPs analysed. The envelope of values corresponds to neutral expectations (with *F*_ST _= 0.078) in the infinite-allele model constructed according to the method of Beaumont and Nichols [21], with a confidence level set to 95%.

Six alleles at five loci (*Lipase, Casein Alpha S1, Interleukin 2, Integrin Beta-1 and Growth Hormone Receptor*) out of 27 were found to lie outside the 95% confidence region of the conditional joint distribution of *FST *and mean heterozygosity (Figure. 1) by *FDIST2 *analysis. They are potentially under selection (P < 0.05) (Tab. 3).

**Figure 2:**
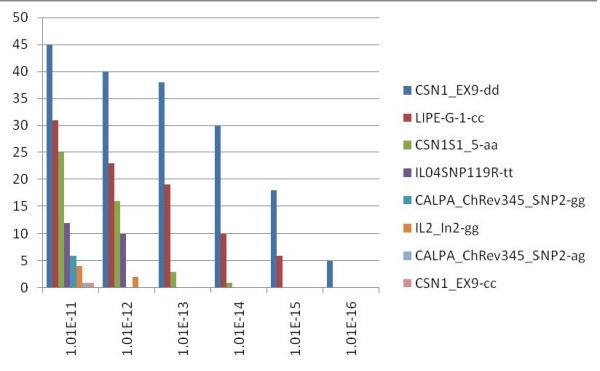
**Plot of significant association models between alleles and environmental parameters**. Histogram of the number of significant association models between genotypes and environmental parameters according to the 6 highest significance levels (Bonferroni correction included) in the analysis. From 1.01E-13 or a confidence level of 99.999999% only *CSN1S1 *and *LIPE *are significantly present. Names of environmental variables are listed in the text. Names of loci in the legend are followed by the genotype [25] associated to the models.

With a significance threshold (ST) set to 95% (corresponding to P < 5.04E-06), SAM detected 16 loci associated with at least one environmental parameter, among which 3 are also identified by *FDIST2 *(*LIPE, CSN1S1-5 and IL2_ln2*). Conversely, *Integrin beta-1 and Growth Hormone Receptor*, identified by *FDIST2*, are not detected by SAM at any confidence level, and are not associated with any environmental parameter. With a ST of 1.01E-13 (confidence level of 99.99999999%, Bonferroni correction included,), SAM identified 3 alleles at 2 loci (*CSN1S1 *and *LIPE*) to be significantly associated with at least one environmental variable. Of the two SNPs analysed in *CSN1S1 *gene, one (*CSN1S1_ex9*) is involved in 38 significant models and is associated with 7 among 10 "families" of environmental parameters (duration of sunshine between may and September; relative humidity in January, May to September, and December; number of days with > 0.1 mm rain per month from may to September, and the yearly mean; temperature from April to September; precipitation from June to September; diurnal temperature range from march to December, and the yearly mean; number of days with ground frost per month from March to November, and the yearly mean). The second SNP in *CSN1S1 *(*CSN1S1-5*) is associated with 3 environmental parameters which are relative humidity in May, the number of days with ground frost per month in April, and diurnal temperature range in October. Finally, one *LIPE *allele is associated with relative humidity from May to September, the coefficient of variation of monthly precipitation in March, diurnal temperature range from March to December, as well as with the yearly mean, and with wind speed from April to May (see Figure 2).

One *CSN1S1_ex9 *allele is the unique involved in the 5 most significant models (1.01E-16 Bonferroni correction included). At this level of significance, it is associated with relative humidity in July and August, with the number of days with > 0.1 mm rain per month in August, with precipitation in August, and with the number of days with ground frost in April.

## Discussion

Rapid adaptation to environments is expected to have shaped at least some of the genetic diversity in marginal European goat breeds [8]. We thus expected that evidence for divergent selection should be detected in goat breeds, as a response to human and environment mediated selection and to migration history [26].

Outlier approaches, in which genes potentially under selection are identified in the extreme tails of empirical distributions, have become a widely used strategy in genome-wide scans for ascertaining selection signatures [11,14,16,17].

Most studies have been conducted using neutral or random markers, like microsatellites or AFLP, however the increasing use of SNPs as a genetic marker due to their rapid discovery rate provides the opportunity to identify genome-wide signatures of selection, as shown in humans [27,28]. There is potentially little power to detect outlier loci when examining populations in pairwise comparisons with biallelic markers [29]. On the contrary, in multi-population analysis the variability in *FST *among loci is reduced facilitating the detection of outlier loci [22]. Lao *et al*. [30] have shown that the frequency distributions of SNPs with ability to detect geographical population structure are most likely shaped by local positive selection rather than by genetic drift. The set of SNPs used for this study have been proven very efficient in detecting geographical patterning (Pariset *et al*, Population analysis of goat breeds from Italy, Albania and Greece reveals precise geographical patterning. Manuscript in preparation).

By calculating *FST *and mean heterozygosis as a measure of genetic differentiation for each locus, we have identified six loci at five genes in goat populations, which have been potentially target of selection according to the distribution of their genetic variation. One of them is related to body size and can be easily selected for by man, two are related to nutritional property of milk (CSN1S1 and LIPE) and they can be both selected by man or by adaptive advantage of the offspring. Interleukin-2 is a gene showing evidence for positive selection in mammals [31] as involved in immune response. The last gene, ITGB1, affects several traits, many of which potentially under selection.

Moreover, by using univariate logistic regression analysis with geographical variables, we could identify 3 of the 5 genes ascertained using *FDIST2*. The combined approach reveals that these three genes are under a selection pressure driven by the environment. One allele at locus *CSN1S1 *is very significantly associated with relative humidity during warm months (July and August), with the frequency of humidity and of precipitation during a warm summer month as well (number of days with > 0.1 mm rain per month and precipitation in August), and with the number of days with ground frost in April. These conditions (warm, wet) are favourable for parasite number to increase over time.

The last association with ground frost is more difficult to interpret, but April could correspond – *grosso modo *– to the moment when the animals come out of the goatshed and this could explain why the association doesn't exist during the other months in winter. *Integrin beta-1 and Growth Hormone Receptor*, identified by *FDIST2*, are not detected by SAM at any confidence level, and are not associated with any environmental parameter. Indeed, such markers can only be detected by their low variance in frequency among populations, and this action requires a dedicated model. Thus, because of their nature they cannot be detected using a regression approach like SAM does. Outlier loci, possibly under selection, can also be driven by other kinds of selection than natural selection. SAM proposes an environment-centered analysis, but there is room left for non-selected environmental effects, and FDIST or BayeScan are able to identify these outliers. This is a reason why Sam can not be used independently, without comparing results with specialized software packages based on theoretical approaches in population genetics, and focused on the analysis of genetic data

While directional selection tends to reduce within-population genetic diversity and to increase among-population differentiation, the reverse is expected under balancing selection, where gene frequencies tend to some equilibrium that maintains polymorphism. In general, Kelley *et al. *[20] found that the outlier approaches determine genes that have been targets of positive selection. Hoffman *et al*. [32] and Minder and Widmer [33] show that it is possible to find candidates for balancing selection using the Beaumont & Nichols [22] approach, but it is difficult because the lower 95% confidence limit is typically close to zero [34].

Among the 6 outlier SNPs, 3 of them had *FST *values lower than the 99% confidence limit in the *FDIST2 *test. We hypothesize that balancing or stabilizing selection may be responsible for these outliers, two of which (*IL2 *and *ITGB2*) have defence-immunity function. Indeed, genes involved in immunity are affected by balancing selection [35] and proteins that perform a defence/immunity function present low *FST *values [11].

In contrast, 3 loci had *FST *values exceeding the 99% confidence limits of *FDIST2 *and two of them were identified also by SAM. These genes might be important in adaptation to different environments and were probably subject to human selection, as well. SAM is able to identify both loci under directional and in stabilizing selection, but requires the comparison with the results produced by *FDIST2 *to differentiate the two types of selection, as is the case for *IL2*. Moreover, SAM can independently validate the *FST *outcomes and find correlations wit geographical variables, pinpointing the probable cause of the selection.

The population divergence methods implemented in *FDIST2 *have been widely used in several papers [7,15,17,32] and have been shown to be quite robust by simulations among various demographic scenarios [34]. Setting the same significance threshold, SAM detects more markers than *FDIST2 *does, but it was shown that when gradually lowering the confidence level in *FDIST2*, the method was also able to identify those additional loci detected by SAM [24]. SAM's sensitivity is not mastered yet, and further studies will be necessary to establish precise relationships between population genomics approaches and these statistical measures of association.

## Conclusion

Data appear congruent by using the two methods for *FST *values exceeding the 99% confidence limits, more markedly than shown in the Joost *et al*. [8] paper where microsatellites were used as markers in sheep populations.

It may surprise that a high percentage of loci appear under selection, but it should be considered that the loci were purposely chosen for influencing potentially selected traits. Moreover, an active trade of livestock was historically present in the studied area [36] and adaptation to novel environment should have been crucial for the survival of imported populations.

The methods of *FST *and SAM can independently detect signatures of selection and therefore can reduce the probability of finding false positives if employed together.

The outlier loci identified in this study can be important because they could indicate adaptive variation in the analysed species, which is characterized by a large range of climatic conditions in the rearing areas and by a history of intense trade, that implies plasticity in adapting to new environments.

## Methods

### Goat breeds

A total of 16 autochthonous goat breeds, originating from Italy, Albania and Greece, were sampled for 30–32 unrelated animals per breed in farms spread over the traditional rearing area of each breed (tab. 2). No more than 3 individuals per farm were sampled to reduce the relationship among animals and to increase the breed representativeness. DNA from a total of 497 blood samples was extracted with a conventional method.

Genotyping was performed by K Biosciences (www Kbioscience.com), using Amplifluor™ (Serologicals™) and Taqman™ (Applied Biosystems™) chemistries using twenty-seven SNP markers, as described in Cappuccio *et al. *[25]. Generally, accuracy greater than 99% was achieved. Quality control criteria were adopted (water as negative control, inter- and intra-plate duplicate testing of a known DNA). Allelic frequency from a total of 13392 genotype assayed was compared to environmental parameters.

### FDIST2 analysis

To detect the effects of selection, the approach used was that proposed by Beaumont and Nichols [22], further developed by Beaumont and Balding [34], and implemented in the *FDIST2 *software . For each locus, the allele frequencies are used to compute *FST *values conditional on heterozygosity and to calculate P-values for each locus.

Each simulation included 32 individuals per population, 16 populations, 27 loci and an expected *FST *of 0.078. This method provides evidence for divergent selection by looking for outliers with *FST *values higher than expected, controlling for heterozygosity [22].

Population datasets were built using 100000 simulations on real data using the coalescent model. Upper and lower confidence limits of 95% quantiles were assumed for conditional joint distribution of *FST *versus mean heterozygosity. Loci showing atypical differentiation behaviour (i.e. *FST*) and lying outside the simulated neutral distribution are then detected as outliers.

### SAM analysis

The Spatial Analysis Method (SAM) described in detail by Joost *et al. *[8] is based on an evaluation of the incidence of spatial coincidence, one of the six concepts of spatial analysis distinguished by Goodchild [37]. Spatial coincidence relates the genetic profile of the organisms studied to the environmental parameters measured at the geographic coordinates of its habitat. The data set used for analysis is in the form of a matrix. Each row of the matrix corresponds to an individual and to the geographic coordinates where it was sampled, while the columns contain a) binary information (1 or 0), relating to the status of the genetic marker (for AFLP markers, 1 or 0 respectively indicate the phenotypes « presence of band » and « absence of band »; for microsatellite and SNP markers, the numbers 1 and 0 respectively, indicate the presence or absence of a given allele at the locus in question) and b) values of environmental parameters at the location in question. Univariate logistic regression analysis are calculated by the SAM Program [38] to determine the degree of association between the frequencies of each allele and the values of the environmental parameters. By calculating the significance of the models generated by all possible pair-wise combinations (allele versus environmental parameter), the markers implicated in the models that emerge as statistically significant can identified. It can be inferred that such loci are likely to influence the process of adaptation to the environment.

### Environmental data

The environmental information used in this study is comprised of altitude and climatic data. Altitude was estimated with the help of the digital elevation model SRTM30 (Shuttle Radar Topography Mission) developed by NASA, which has a resolution of 30 arc seconds. The climatic data described in Table 1 are based upon grids of 10 minutes of resolution (equivalent to approximately 12 km at the latitude of Switzerland). They are presented in the form of 9 monthly averages and an annual average. These variables characterise continental regions during the time period between 1961 and 1990 [39]. The data have been collected by the Climatic Research Unit (CRU) in Norwich, UK. For this study, monthly variables were separately analysed in order to take account of the seasonality of kidding [40-42].

**Table 1:** SNPs showing simulated *FST *< sample *FST *after *FDIST2 *analysis.

**SNP**	**locus**	**sample He**	**sample *FST***,	**test statistic**	P*
*LIPE*	*lipase*	0.50678	0.26101	3.95642	0.99995
*GHR*	*growth hormone receptor*	0.50032	0.01206	-2.77215	0.00350
*CSN1-5*	*casein alphaS1*	0.50540	0.15638	2.06217	0.97596
*ITGB1*	*integrin beta-1*	0.46289	0.02699	-2.01899	0.02662
*IL2_5*	*interleukin 2*	0.50477	0.14332	1.77708	0.95433
*IL2 IN2*	*interleukin 2*	0.03265	0.00721	-5.00000	0.00000

*P(simulated *FST *< sample *FST*)

**Table 2:** Breeds analysed, country of origin and their sample sizes (N).

**Breed**	**Origin**	**N**
Argentata dell'Etna	Italy	31
Bionda dell'Adamello	Italy	31
Camosciata	Italy	31
Capore	Albania	31
Dukati	Albania	31
Girgentana	Italy	32
Greek goat	Greece	31
Grigia molisana	Italy	31
Hasi	Albania	31
Liquenasi	Albania	31
Mati	Albania	30
Muzhake	Albania	31
Orobica	Italy	31
Sarda	Italy	31
Skopelos	Greece	31
Valdostana	Italy	31

List of environmental variables considered in SAM analysis. Yearly means and monthly values were used for these climatic variables (a total of 118 environmental parameters).

**Table 3:** Environmental variables considered in SAM analysis

**Variable**	**Description**
Altitude	DEM SRTM30, NASA
DTR	Diurnal Temperature Range in °C
FRS	Number of days with ground frost
PR	Precipitation in mm/month
PRCV	Coefficient of variation of monthly precipitation in %
REH	Relative humidity (%)
SUN	Percent of maximum possible sunshine (percent of daylength)
TMP	Mean temperature in °C
RDO	Number of days with > 0.1 mm rain per month
WIND	Windspeed in m/s 10 meters above the ground

List of environmental variables considered in SAM analysis. Yearly means and monthly values were used for these climatic variables (a total of 118 environmental parameters).

## Competing interests

The authors declare that they have no competing interests.

## Authors' contributions

LP: designed the study, performed the *FST *analysis, and drafted the manuscript. SJ: developed the SAM approach, carried out the relative computations, and formulated parts of the paper. PAM: leader of he Econogene project, provided DNA samples and guidance for the project. AV: participated in developing ideas, in supervision and revision of the manuscript.
